# Nomogram prediction model of postoperative pneumonia in patients with lung cancer: A retrospective cohort study

**DOI:** 10.3389/fonc.2023.1114302

**Published:** 2023-02-23

**Authors:** Fan Jin, Wei Liu, Xi Qiao, Jingpu Shi, Rui Xin, Hui-Qun Jia

**Affiliations:** ^1^ Department of Anesthesiology, The Fourth hospital of Hebei Medical University, Shijiazhuang, Hebei, China; ^2^ Department of Anesthesiology, Zhuji People’s Hospital, Shaoxing, Zhejiang, China

**Keywords:** postoperative pneumonia, nomogram, lung cancer, risk factors, thoracic surgery

## Abstract

**Background:**

The prediction model of postoperative pneumonia (POP) after lung cancer surgery is still scarce.

**Methods:**

Retrospective analysis of patients with lung cancer who underwent surgery at The Fourth Hospital of Hebei Medical University from September 2019 to March 2020 was performed. All patients were randomly divided into two groups, training cohort and validation cohort at the ratio of 7:3. The nomogram was formulated based on the results of multivariable logistic regression analysis and clinically important factors associated with POP. Concordance index (C-index), receiver operating characteristic (ROC) curve, calibration curve, Hosmer-Lemeshow goodness-of-fit test and decision curve analysis (DCA) were used to evaluate the predictive performance of the nomogram.

**Results:**

A total of 1252 patients with lung cancer was enrolled, including 877 cases in the training cohort and 375 cases in the validation cohort. POP was found in 201 of 877 patients (22.9%) and 89 of 375 patients (23.7%) in the training and validation cohorts, respectively. The model consisted of six variables, including smoking, diabetes mellitus, history of preoperative chemotherapy, thoracotomy, ASA grade and surgery time. The C-index from AUC was 0.717 (95%CI:0.677-0.758) in the training cohort and 0.726 (95%CI:0.661-0.790) in the validation cohort. The calibration curves showed the model had good agreement. The result of DCA showed that the model had good clinical benefits.

**Conclusion:**

This proposed nomogram could predict the risk of POP in patients with lung cancer surgery in advance, which can help clinician make reasonable preventive and treatment measures.

## Introduction

As the Global Cancer Statistics reported in 2020, lung cancer has become the second most common cancer and the highest rate of cancer-related death (1). The treatments of lung cancer mainly include radiotherapy, chemotherapy, surgery, targeted therapy, immunotherapy. However, surgical resection is still an effective and safe intervention for patients with lung cancer.

Unfortunately, the postoperative pulmonary complications (PPCs) of lung cancer surgery are still common problems and major challenges for patient recovery. And postoperative pneumonia (POP) has become the most common PPCs (2, 3). Several studies have found that the rate of POP in lung cancer patients is about 2%-25% (4–7). And POP could significantly prolong the length of hospitalization, increase hospitalization expense, and even increase perioperative mortality (4, 8–10). Thus, early identification of risk factors associated with POP among patients with lung cancer could be beneficial to forecast the risk of POP in advance.

Potential risk factors for POP existed throughout the whole perioperative period (11). Preoperative factors mainly include elderly, smoking, pulmonary function, comorbidities and nutritional status (12, 13). Intraoperative factors include surgery types, duration of surgery, anesthesia types and ventilation mode (6, 14). Postoperative factors include acute pain and other complications (15, 16). A retrospective observational cohort (n=7479) found that elderly, preoperative pulmonary infection, atrial fibrillation, obesity, and alcohol might be associated with POP among lung cancer patients (10). Deguchi et al. (5) found preoperative asthma might also be independently associated with POP in lung cancer patients. And Yendamuri et al. (17) found age >75 years, male, thoracotomy, COPD and American Society of Anesthesiologists (ASA) ≥III might be potential risk factors after analyzing a range of patients (n=12562) who underwent pulmonary lobectomy. However, most of the present studies on POP in lung cancer patients had only identified risk factors for POP, studies about prediction models for POP were very limited.

Although there had a model to predict POP for elderly patients who underwent video-assisted thoracoscopic surgery for lung cancer, the data was collected from 2012 to 2019 and other variables associating with POP such as anesthesia types, anesthetics and surgery types were scarce (4). Thus, the development of a model after fully considering the preoperative and intraoperative variables to forecast the risk of POP in advance would be beneficial to patients.

Therefore, the purpose of this study was to develop and validate a model to predict POP in patients undergoing lung cancer surgery and investigate risk factors for POP so that reasonable preventive and treatment measures could be made earlier.

## Methods

### Study design

This is a retrospective cohort study, which has been approved by Clinical Research Ethics Committee of the Fourth Hospital of Hebei Medical University (No:2022KS024), Shijiazhuang, Hebei Province, China (Chairperson Prof Hongtao He) on 28 July 2022. The informed consent was exempted with the approval of the local ethics committee. All patients were randomly separated into training and validation cohorts at the ratio of 7:3 which was similar to other studies (18–20). The training cohort was conducted to develop prediction model, while both training and validation cohorts were used to verify the predictive ability of the model.

### Participants

We retrospectively analyzed patients who underwent thoracic surgery from September 2019 to March 2020 at the Fourth Hospital of Hebei Medical University. The inclusion criteria were patients aged ≥ 18 years with pathology diagnosis of lung cancer and underwent surgery. Patients were excluded if they met one or more of following criteria: 1) preoperative pneumonia diagnosed by computed tomography (CT), 2) bilateral pulmonary resection, 3) reoperation within 30 days, 4) admitted to ICU after surgery, 5) missing data.

### Perioperative management

All patients received general anesthesia, either alone or in combined with regional nerve block (including paravertebral nerve block, epidural anesthesia, and intercostal nerve block.) according to the type of surgery. Patients underwent lobectomy or sublobectomy according to surgeon’s comprehensive evaluation based on patient’s condition.

Anesthesia induction used propofol and/or etomidate, sufentanil, and rocuronium or cisatracurium. Anesthesia maintenance used sevoflurane or propofol combined with remifentanil or sufentanil. Rocuronium or cisatracurium was used to maintain muscle relaxation. Supplemental drugs such as flurbiprofen axetil were administered when necessary. The aim was to maintain BIS 40-60, blood pressure within 20% of baseline, and temperature 36-37°C.

Double-lumen endotracheal tube of sizes Ch33-39 was used for lung isolation according to patient height. The ventilation mode was volume control mode with 6-8 ml/kg of tidal volume (TV) during two-lung ventilation and 5-6 ml/kg during one-lung ventilation (OLA), and 0-5 cmH_2_O of positive end-expiratory pressure (PEEP), and 12-20 breaths/min of respiratory rates. The aim was to maintain P_ET_CO_2_ 35-45 mmHg and SpO_2_ ≥92%. At the end of anesthesia, neostigmine was used to antagonize muscular relaxant before extubation.

Fluid infusion was administrated with crystalloid at a rate of 4–6 mL/kg^-1^h^-1^. Colloids or blood product was used according to anesthesiologist’s comprehensive evaluation based on patient’s condition. Patient-controlled intravenous analgesia was used after surgery for postoperative analgesia to maintain numeric rating scales (NRS) ≤ 3 scores.

### Data collection

We collected following variables, including: 1) basic demographics such as age, sex, history of smoking, body mass index (BMI), preoperative chemotherapy, history of lung surgery; 2) preoperative comorbidities containing hypertension, chronic obstructive pulmonary disease (COPD), asthma, diabetes, coronary heart disease, arrhythmia; 3) preoperative laboratory testing including Hemoglobin, serum albumin, Serum glucose; 4) preoperative pulmonary function including forced vital capacity rate of one second(FEV_1_/FVC), diffusion capacity for carbon monoxide of the lung(D_L_CO); 5) surgery related characteristics including surgery types, surgery extent, surgery sides, duration of surgery; 6) anesthesia related characteristics including ASA grade, anesthesia types, use of flurbiprofen axetil, use of colloid, allogenic blood transfusion, Input per unit of time (ml·kg^-1^·h^-1^). Smoking was defined as smoking index ≥ 400. Duration of surgery was defined as the time interval between skin incision and suture. Input per unit of time was equal to total input divided by duration of surgery and actual weight.

### Diagnosis of pneumonia

POP was occurred during hospitalization, which defined as follows (21): patient has received antibiotics for a suspected respiratory infection and met one or more of the following criteria: 1) new or changed sputum, 2) new or changed lung opacities, 3) fewer (>38.3°C), 4) white blood cell count >12×10^9^/L.

### Statistical analysis

The normal distribution data were present as mean ± standard deviation (SD) and compared by the independent sample t-test, while the non-normal distribution data were present as median(*Q*_1_, *Q*_3_) and compared by the Wilcoxon test. And the categorical data were present as number and percentages, and compared by the Chi-square test.

The Least Absolute Shrinkage and Selection Operator (LASSO) logistic regression with 5-fold cross-validation was used to adjust the parameter lambda to screen the variables. And the lambda corresponding to the minimum mean square error was used for selecting variables. The multivariate logistic regression analysis was used to analyze characteristic variables selected by LASSO regression to explore the independent risk factors associated with POP. A nomogram was built according to the independent risk factors and clinically important factors associated with POP.

The AUROC and C index were used to measure the discrimination ability according to the data from training and validation cohorts. And the calibration ability was measured by the calibration curve and Hosmer-Lemeshow goodness-of-fit test. The clinical benefit was measured by the decision curve analysis (DCA). Statistical analysis was performed with R software (version 3.5.3; https://www.R-project.org). A p<0.05 with two sides was considered statistical significance.

## Results

### Participants

**Figure 1:**
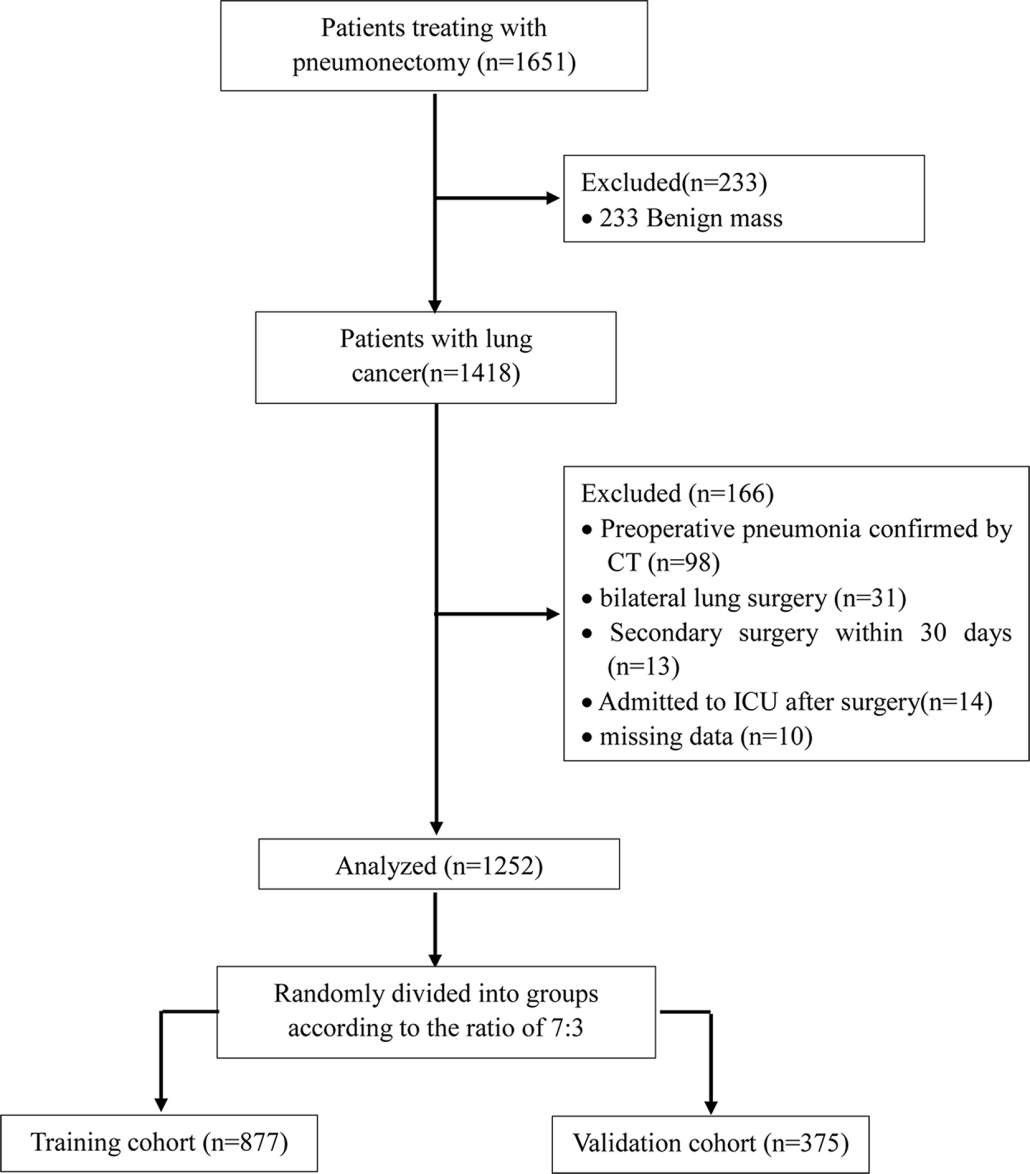
Flow chart of patients screening and recruitment.

We initially screened 1651 patients who underwent thoracic surgery from September 2019 to March 2020 (**Figure 1**). Of these, 233 patients were benign mass and the rest of 1418 patients were included in the study. After data collection, 166 patients were removed from the final analysis: 98 patients had preoperative pneumonia confirmed by CT; 31 patients required bilateral pulmonary resection; 13 patients required reoperation within 30 days; 14 patients admitted to ICU after surgery; 10 patients had missing data. Finally, a total of 1252 patients were admitted into the present study. The training cohort had 877 patients who were aged 60.0 ± 9.4 years. The validation cohort consisted of 375 patients who were aged 59.5 ± 9.7 years. The incidence of POP was 22.9% in the training cohort, 23.7% in the validation cohort and 23.2% among all patients. All variables expect for Hemoglobin (*P*<0.05) were no statistically significant differences between two groups (other *P* > 0.05) (**Table 1**).

**Table 1:** Patient Basic Characteristics.

Variables	Total (n=1252)	Training (n=877)	Validation (n=375)	*P*-Value
Age, (y, mean ± SD)	59.8 ± 9.5	60.0 ± 9.4	59.5 ± 9.7	0.452
Sex, n (%)				0.088
Male	677 (54.1)	488 (55.6)	189 (50.4)	
Female	575 (45.9)	389 (44.4)	186 (49.6)	
BMI (kg/m^2^, mean ± SD)	25.2 ± 3.3	25.2 ± 3.3	25.1 ± 3.2	0.868
Comorbidities, n (%)				
COPD	48 (3.8)	35 (4.0)	13(3.5)	0.658
Asthma	6 (0.5)	5 (0.6)	1 (0.3)	0.675
Hypertension	448 (35.8)	313 (35.7)	135 (36.0)	0.916
Coronary heart disease	100 (8.0)	72 (8.2)	28 (7.5)	0.657
Arrhythmia ^a^	53 (4.2)	41 (4.7)	12 (3.2)	0.235
Diabetes	156 (12.5)	104 (11.9)	52 (13.9)	0.324
ASA, n (%)				0.430
II	1070 (85.5)	745 (84.9)	325 (86.7)	
III	182 (14.5)	132 (15.1)	50 (13.3)	
Smoking ^b^, n (%)	325 (26.0)	224 (25.5)	101 (26.9)	0.607
Alcohol, n (%)	232 (18.5)	155 (17.7)	77 (20.5)	0.233
History of lung surgery, n (%)	16 (1.3)	14 (1.6)	2 (0.5)	0.171
Preoperative chemotherapy, n(%)	66 (5.3)	51 (5.8)	15 (4.0)	0.188
FEV_1_/FVC, n (%)				0.177
<0.7	232 (18.5)	154 (17.6)	78 (20.8)	
≥0.7	1020 (81.5)	723 (82.4)	297 (79.2)	
D_L_CO, n (%)				0.823
<80%	225 (18.0)	159 (18.1)	66 (17.6)	
≥80%	1027 (82.0)	718 (81.9)	309 (82.4)	
Hemoglobin, (g/L, *M*(*Q*_1_, *Q*_3_))	139.0 (129.0,149.0)	138.0 (128.0,148.0)	141.0 (131.0, 150.0)	0.024
Serum albumin, (g/L, mean ± SD)	43.5 ± 4.1	43.4 ± 3.8	43.8 ± 4.7	0.110
Serum glucose, (mmol/L, *M*(*Q*_1_, *Q*_3_))	5.1 (4.8,5.7)	5.1 (4.8,5.7)	5.2 (4.8,5.9)	0.140
Surgery type, n (%)				0.600
VATS	1106 (88.3)	772 (88.0)	334 (89.1)	
Thoracotomy	146 (11.7)	105 (12.0)	41 (10.9)	
Surgery extent, n (%)				0.840
Sublobectomy ^c^	201 (16.1)	142 (16.2)	59 (15.7)	
Lobectomy ^d^	1051 (83.9)	735 (83.8)	316 (84.3)	
Surgery side, n (%)				0.238
center side	493 (39.4)	336 (38.3)	157 (41.9)	
Right side	759 (60.6)	541 (61.7)	218 (58.1)	
Anesthesia type, n (%)				0.504
GA only	328 (26.2)	225 (25.7)	103 (27.5)	
GA + RA ^e^	924 (73.8)	652 (74.3)	272 (72.5)	
Use of flurbiprofen axetil, n (%)	389 (31.1)	269 (30.7)	120 (32.0)	0.642
Input per unit of time, (ml·kg^-1^·h^-1^, *M*(*Q*_1_, *Q*_3_))^f^	5.3 (4.3,6.2)	5.2 (4.3,6.3)	5.3 (4.2,6.2)	0.746
Use of colloid, n (%)	979 (78.2)	696 (79.4)	283 (75.5)	0.126
Allogenic blood transfusion, n (%)	40 (3.2)	30 (3.4)	10 (2.7)	0.487
Duration of surgery, (h, *M*(*Q*_1_, *Q*_3_))	2.7 (2.1,3.3)	2.7 (2.1,3.4)	2.7 (2.1,3.3)	0.468
POP, n (%)	290 (23.2)	201 (22.9)	89 (23.7)	0.754

Data are mean ± SD or median (Q_1_, Q_3_) or number (%). BMI, Body Mass Index; COPD, Chronic Obstructive Pulmonary Disease; ASA, American Society of Anesthesiologists; FEV_1_/FVC, Forced Vital Capacity rate of one second; D_L_CO, Diffusion Capacity for Carbon Monoxide of the Lung; VATS, Video-assisted Thoracoscopic Surgery; GA, general anesthesia; RA, Regional nerve block; POP, Postoperative Pneumonia.

^a^Including atrial fibrillation, atrial flutter, and atrioventricular block.

^b^Smoking was defined as smoking index ≥ 400.

^c^Including lung wedge resection and segmentectomy.

^d^Including Lobectomy and pneumonectomy.

^e^including epidural anesthesia, paravertebral nerve block, and intercostal nerve block.

^f^Input per unit of time =total input(ml)÷duration of surgery(h) ÷actual weight(kg).

### Development of prediction model

**Figure 2:**
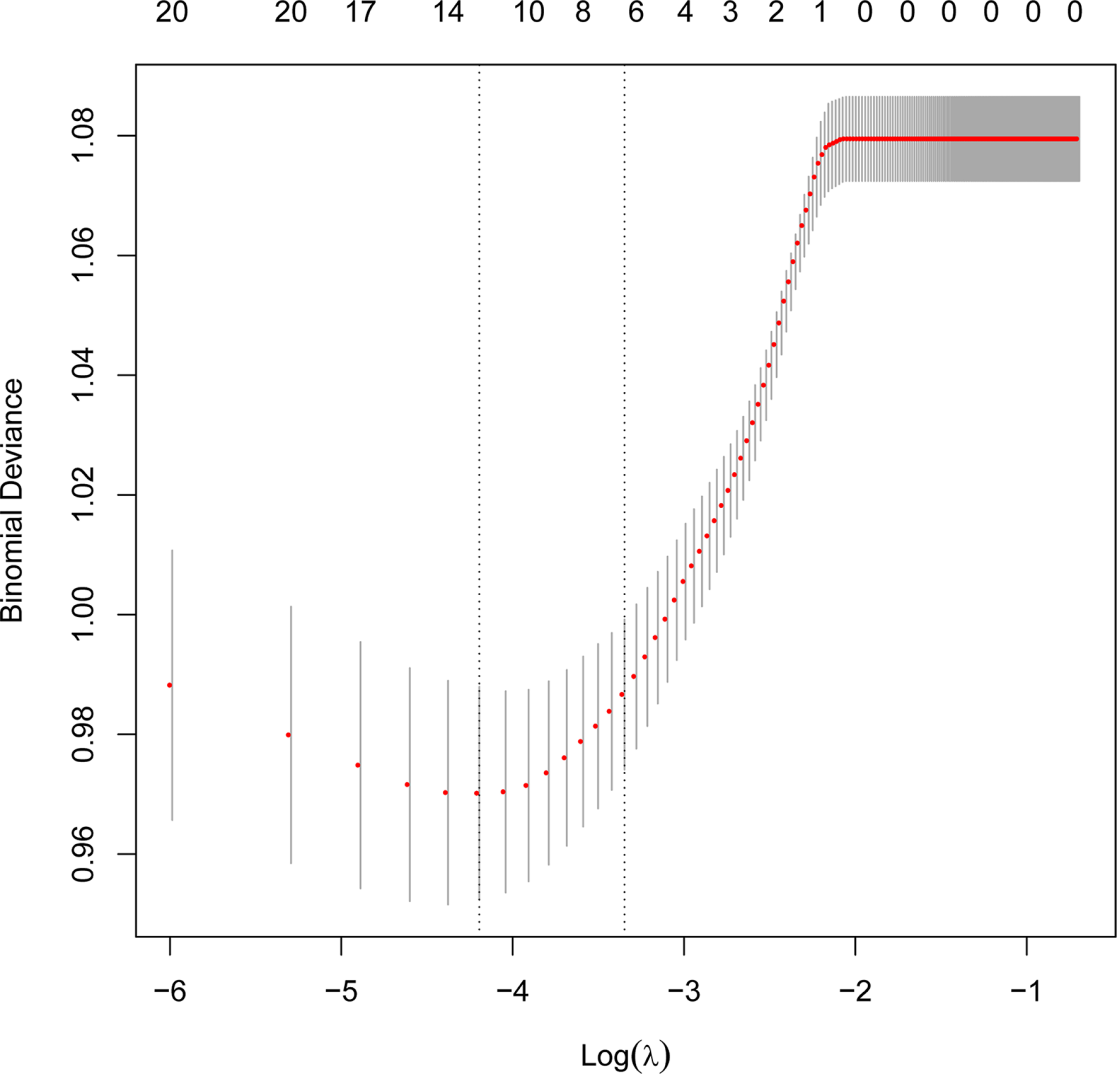
Perioperative variables selection using the Least Absolute Shrinkage and Selection Operator (LASSO) regression.

The protential risk factors of POP were selected by LASSO regression (**Figure 2**). The coefficients of relatively irrelevant variables were minimized to 0 and subsequently were excluded according to the value of lambda. The LASSO regression showed the optimal value of lambda was 0.015 (**Table 2**). And 12 non-zero representative variables were remained, including age, history of smoking, diabetes, preoperative chemotherapy, FEV_1_/FVC, D_L_CO, surgery type, ASA grade, use of flurbiprofen axetil, use of colloid, input per unit of time and duration of surgery (**Table 2**).

**Table 2:** Coefficients of the LASSO Regression.

Variables	Coefficients	Lambda. Min
Age	0.008	0.015
Smoking	0.207	
Diabetes	0.370	
Preoperative chemotherapy	1.160	
FEV_1_/FVC	0.146	
D_L_CO	0.018	
Surgery type	0.496	
ASA	0.445	
Use of flurbiprofen axetil	0.028	
Use of colloid	-0.126	
Input per unit of time	-0.026	
Duration of surgery	0.336	

ASA, American Society of Anesthesiologists; FEV_1_/FVC, Forced Vital Capacity rate of one second; D_L_CO, Diffusion Capacity for Carbon Monoxide of the Lung.

On the multivariate logistic regression analysis, there were five variables independently associated with POP, including diabetes (OR=1.838; 95%CI:1.110-3.001); preoperative chemotherapy (OR=3.997; 95%CI:2.014-8.093); Thoracotomy (OR=1.891; 95%CI:1.126-3.138); ASA grade (OR=1.760; 95%CI:1.105-2.780); and duration of surgery (OR=1.486; 95%CI:1.268-1.750) (**Table 3**).

**Table 3:** Multivariate Logistic Regression Analysis of POP Based on Data in the Training Cohort.

Variables	β Coefficient	OR (95%CI)	*P*-Value
Diabetes (Y/N)	0.608	1.838 (1.110-3.001)	0.016
Preoperative chemotherapy(Y/N)	1.386	3.997 (2.014-8.093)	<0.001
Surgery type(Thoracotomy/VATS)	0.637	1.891 (1.126-3.138)	0.015
ASA(III/II)	0.565	1.760 (1.105-2.780)	0.016
Duration of surgery (h)	0.396	1.486 (1.268-1.750)	<0.001

POP, postoperative pneumonia; OR, odds ratio; CI, confidence interval; ASA, American Society of Anesthesiologists. Y, Yes; N, No.

Although multivariate logistic regression analysis showed smoking was not an independent factor, the coefficient of smoking was larger according to LASSO regression. We thought smoking might influence the incidence of POP. So we used five independent risk factors and smoking to draw a nomogram to develop a POP prediction model. The dynamic nomogram of POP is available online (https://lungcancersurgery.shinyapps.io/DynNomapp/). The code of dynamic nomogram is presented in **Supplement Material**.

### Validation of prediction model

**Figure 3:**
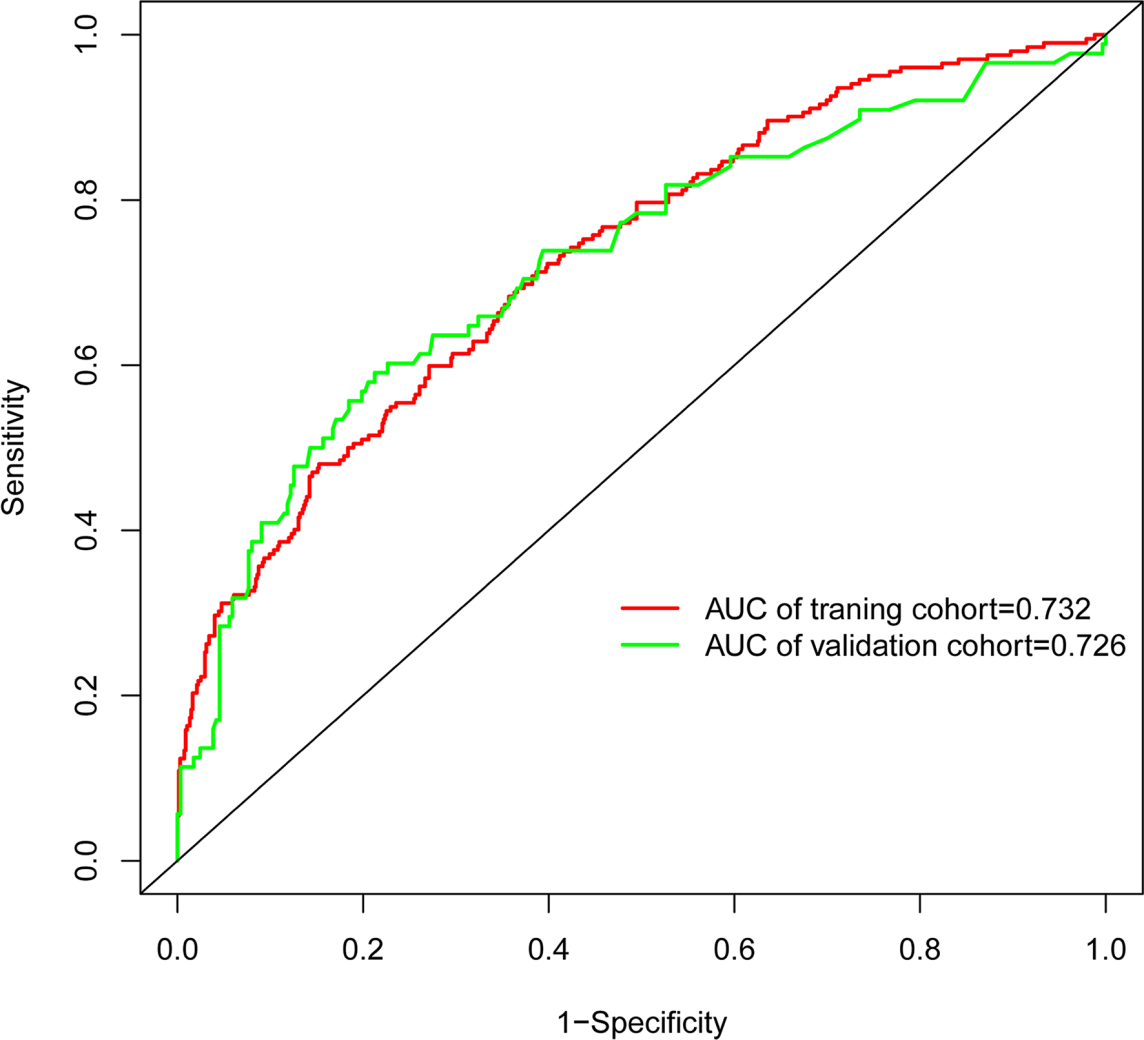
The receiver operating characteristic (ROC) curve of POP risk nomogram.

**Figure 4:**
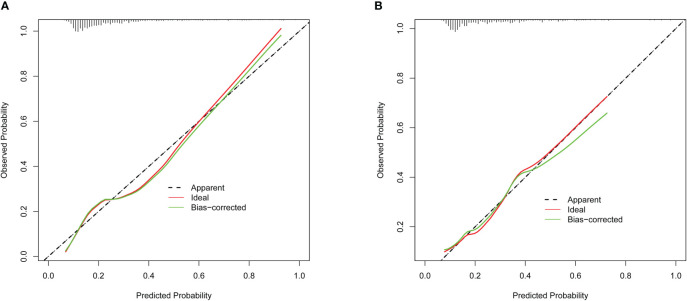
The calibration curve of POP risk nomogram. **(A)** Calibration curve in the training cohort (n = 877). **(B)** Calibration curve in the validation cohort (n = 375).

**Figure 5:**
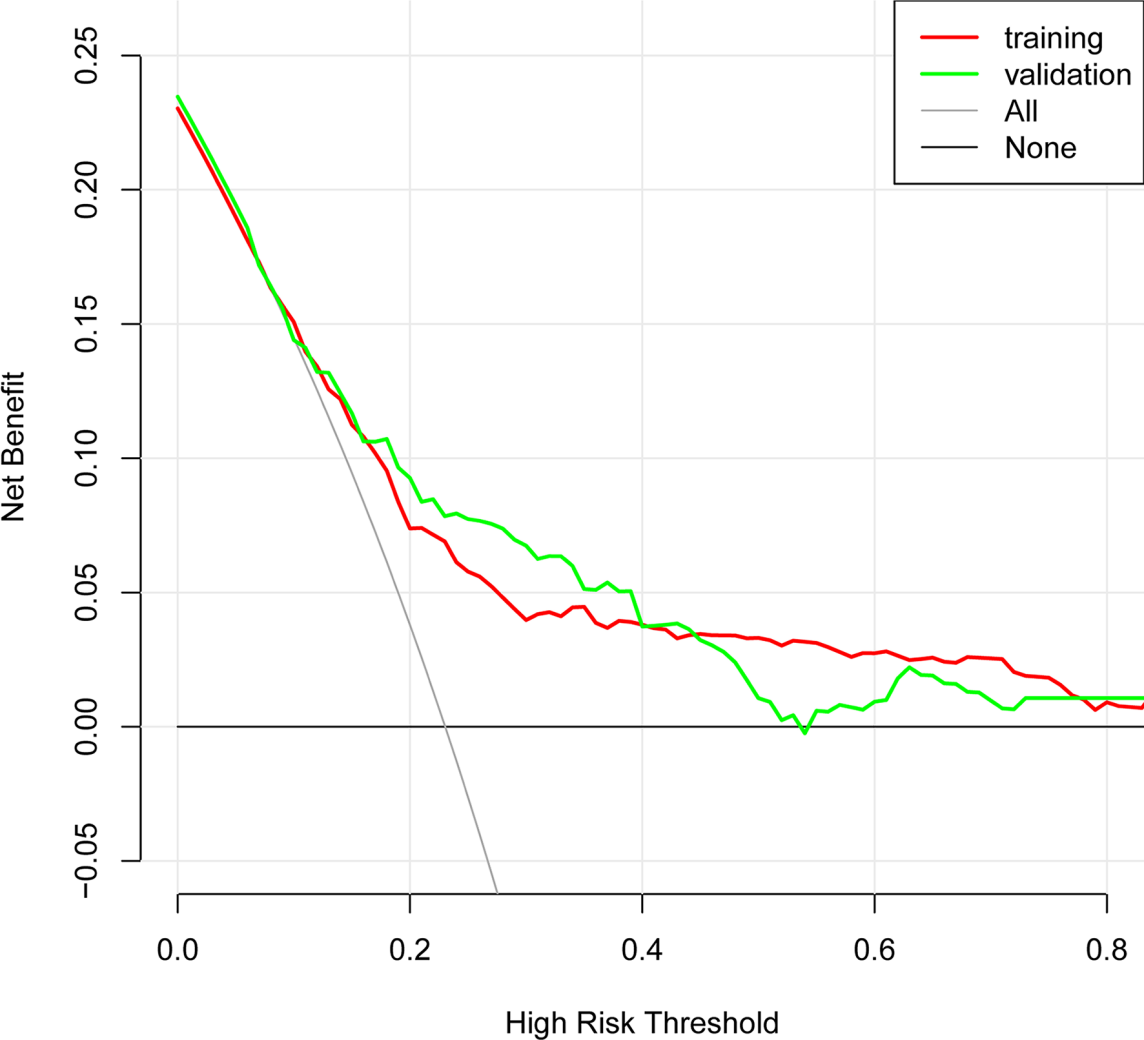
The decision curve analysis (DCA) of POP risk nomogram.

In the present, the uncorrected C index was 0.717 (95%CI:0.677-0.758) and bootstrap-corrected C index was 0.710 in the training cohort, while the uncorrected C index was 0.726 (95%CI: 0.661-0.790) and bootstrap-corrected C index was 0.709 in the validation cohort (**Figure 3**). These results showed the nomogram had good accuracy in distinguishing patients with and without POP. Besides, the calibration curve showed good consistency on the presence of POP between prediction by the nomogram and results of actual clinical data (**Figure 4**), which demonstrated by Hosmer-Lemeshow goodness-of-fit test both in the training and validation cohorts (both *P >*0.05). At the same time, the decision curve analysis showed a positive net benefit when the predicted probability threshold is 0%-80%, indicating this nomogram had good clinical benefit (**Figure 5**).

## Discussion

This study developed and validated a nomogram to accurately forecast the risk of POP in patients who underwent lung cancer surgery. This model included six preoperative and intraoperative variables, including smoking, diabetes, preoperative chemotherapy, surgery types, ASA grade and duration of surgery, which predicted well as demonstrated by the uncorrected C index values of 0.717 and 0.726 in the training and validation cohorts. At the same time, the calibration curves showed good consistency between prediction and actual observation, respectively, and the decision curve analysis indicated this nomogram had good clinical benefit.

LASSO regression is a common method for variable selection in fitting high-dimensional generalized linear and has been widely used in clinical research (22, 23). The LASSO method selects variables *via* minimizing the coefficients of relatively irrelevant variables to 0 and subsequently removing these variables by constructing a penalty function, which effectively avoids the overfitting and makes the model more refined (24, 25). So we used the LASSO regression for variable selection in this study.

The nomogram we used is a superior visual tool and has been widely used in the clinical practice, which has several advantages. Firstly, the nomogram could transform predictive model into a single estimate of probability according to patient’s characteristics, making model simple to understand (26). Secondly, the scoring system has high precision and good stability characteristics in predicting results (27, 28). Therefore, we used nomogram to build the visual model to help clinicians to stratify patients and develop individual clinical treatment strategies according to patient’s conditions.

Evaluating the characteristics of the predictive model from multiple perspectives and selecting the optimal model could help promotion and application of the model (29). The calibration ability is model’s capability to demonstrate the consistency between the actual observed and the prediction by the model, which is one of the best indicators to reflect predictive performance of the model (29). Therefore, we used calibration curve and Hosmer-Lemeshow goodness-of-fit test to evaluate calibration ability of this model. And good agreements between prediction and actual observation were supported by Hosmer-Lemeshow goodness-of-fit test. However, good calibration couldn’t perfectly distinguish patients with or without POP. The ROC curve and C index had certain advantages in measuring discrimination ability of the model (30). And the results showed that the model could distinguish patients with and without POP. Furthermore, we used the decision curve analysis and net benefit to evaluate the clinical benefit of the model (31). The results suggested that using this model to assist clinical treatment strategies might help improve patient prognosis.

In the POP risk estimation nomogram, preoperative chemotherapy, thoracotomy, ASA and duration of surgery have been confirmed to increase the risk of POP (17, 32, 33). This study showed that above factors were also independent risk factors associated with POP in lung cancer patients. In addition, we illustrated that diabetes was associated with POP in patients with lung cancer surgery.

In previous reports, diabetes was associated with POP after surgery (34). The incidence of POP in type 2 diabetes mellitus patients was 21% higher than non-diabetic patients (35). In the present study, we found the risk of POP was higher in diabetes patients (OR=1.838; 95%CI:1.110-3.001) after lung cancer surgery. The potential mechanisms were diabetes could destroy innate immunity in pulmonary, impair pulmonary function and reduce cardiorespiratory fitness making patients more susceptible to infections (36).

However, this study still has some limitations. At first, the bias of patient selection could not be entirely avoided because it was a single-center retrospective study. However, we screened patients through strict inclusion and exclusion criteria, which could reduce population homogeneity to some extent. Secondly, the definition of POP in our study might be generic and sensitive to diagnose pneumonia, which made the incidence of POP a little higher than other studies. However, we used the same criteria in diagnosing pneumonia in our study which could make result reliable to some extent. Thirdly, intraoperative respiratory parameters, such as tidal volume, minute ventilation, PEEP were not included in our study because these variables could not be collected. However, some studies had found that intraoperative ventilation strategy might not be associated with postoperative pulmonary complications (37, 38). Finally, some postoperative variables, such as postoperative pain, postoperative aerosolized inhalation might influence the rate of POP among patients after lung cancer surgery, but these variables were not analyzed in the study because we aimed at predicting POP through preoperative and intraoperative variables rather than postoperative variables.

## Conclusion

This study developed and validated a predictive model representing by the nomogram to quantify the risk of POP in patients with lung cancer surgery. This model showed good discrimination ability, calibration ability and clinical benefit which could help make better prevention and individual treatment strategies in advance.

## Data availability statement

The code of dynamic nomogram in the study is included in the article/**Supplementary Material**. Further inquiries can be directed to the corresponding author.

## Ethics statement

The study involving human participants was reviewed and approved by Ethics Committee of The Fourth hospital of Hebei Medical University. Written informed consent for participation was not required for this study in accordance with the national legislation and the institutional requirements.

## Author contributions

JF: This author helped in data acquisition, data analysis, and manuscript drafting. LW: This author helped in data acquisition, and data analysis. XQ: This author helped in data acquisition, and data analysis. JS: This author helped in data acquisition. RX: This author helped in data acquisition. H-QJ: This author helped in concept and design, data analysis, data interpretation, revision of the manuscript, and final approval of submission. All authors contributed to the article and approved the submitted version.
