# Supplementary material for: Nomogram prediction model of postoperative pneumonia in patients with lung cancer: A retrospective cohort study
Source: Front Oncol. 2023 Feb 23;13:1114302. doi: 10.3389/fonc.2023.1114302 (PMC9996165; doi:10.3389/fonc.2023.1114302)
Supplement: Supplementary file 1 [file DataSheet_1.docx]

library(rms)

library(DynNom)

rm(list=ls())

lun<-read.csv("lun.csv")

str(lun)

lun$Smoking<- factor(lun$Smoking,levels=c("0","1"),labels=c("No","Yes"))

lun$Diabetes<- factor(lun$Diabetes,levels=c("0","1"),labels=c("No","Yes"))

lun$Chemotherapy<- factor(lun$Chemotherapy,levels=c("0","1"),labels=c("No","Yes"))

lun$Surgery_type<- factor(lun$Surgery_type,levels=c("0","1"),labels=c("VATS","Thoracotomy"))

lun$ASA<- factor(lun$ASA,levels=c("2","3"),labels=c("Ⅱ","Ⅲ"))

dd<-datadist(lun)

options(datadist = "dd")

(formul <-as.formula(POP~Smoking+Diabetes+Chemotherapy+Surgery_type+ASA+Surgery_time_h

))

fit2<-glm(formul,data=lun,family = binomial())

DynNom(fit2,data=lun,DNtitle = "Nomogram",DNxlab = "Probability")

DNbuilder(fit2)

install.packages('rsconnect')

rsconnect::setAccountInfo(name='lungcancersurgery',

token='E2AB7FD198A8F51F34497DF6DDA3D586',

secret='yjpb2ZvMrhDH24k/FT6vJG66bz25uKMGm6nEUkic')

ui = bootstrapPage(fluidPage(

titlePanel('Dynamic Nomogram of Postoperative Pneumonia in Patients with Lung Cancer'),

sidebarLayout(sidebarPanel(uiOutput('manySliders'),

uiOutput('setlimits'),

actionButton('add', 'Predict'),

br(), br(),

helpText('Press Quit to exit the application'),

actionButton('quit', 'Quit')

),

mainPanel(tabsetPanel(id = 'tabs',

tabPanel('Graphical Summary', plotlyOutput('plot')),

tabPanel('Numerical Summary', verbatimTextOutput('data.pred')),

tabPanel('Model Summary', verbatimTextOutput('summary'))

)

)

)))
